# Standardized Edible Bird’s Nest Extract Prevents UVB Irradiation-Mediated Oxidative Stress and Photoaging in the Skin

**DOI:** 10.3390/antiox10091452

**Published:** 2021-09-13

**Authors:** Ok-Kyung Kim, Dakyung Kim, Minhee Lee, Seong-Hoo Park, Wakana Yamada, Sangwon Eun, Jeongmin Lee

**Affiliations:** 1 Division of Food and Nutrition and Human Ecology Research Institute, Chonnam National University, Gwangju 61186, Korea; 20woskxm@jnu.ac.kr; 2 Department of Medical Nutrition, Kyung Hee University, Yongin 17104, Korea; k4kyung@naver.com (D.K.); miniclsrn@khu.ac.kr (M.L.); phoo3166@khu.ac.kr (S.-H.P.); 3 Oryza Oil & Fat Chemical Co., Ltd., Ichinomiya 493-8001, Japan; kaihatsu@mri.biglobe.ne.jp; 4 R&D Division, Daehan Chemtech Co. Ltd., Seoul 01811, Korea; info@dhchemtech.com; 5 Research Institute of Clinical Nutrition, Kyung Hee University, Seoul 02447, Korea

**Keywords:** edible bird’s nest, ultraviolet B, oxidative stress, skin photoaging

## Abstract

We investigated whether standardized edible bird’s nest extract (BNE-PK) can prevent ultraviolet B (UVB) irradiation-mediated oxidative stress and photoaging in the skin using in vitro and in vivo models. BNE-PK increased skin hydration by hyaluronic acid synthesis and activation of ceramide synthase in UVB-irradiated hairless mice and HaCaT cells. Furthermore, BNE-PK suppressed melanogenesis by down-regulation of the cAMP/PKA/CREB/MITF/TRP-1/TRP-2/tyrosinase pathway in UVB-irradiated hairless mice and 3-isobutyl-1-methylxanthine (IBMX)-treated B16F10 cells. In UVB-irradiated hairless mice, BNE-PK attenuated the wrinkle formation-related JNK/c-FOS/c-Jun/MMP pathway and activated the TGF-βRI/SMAD3/pro-collagen type I pathway during UVB-mediated oxidative stress. Based on these findings, our data suggest that BNE-PK may potentially be used for the development of effective natural anti-photoaging functional foods for skin health.

## 1. Introduction

The skin is one of the important organs that performs biochemical and physical defense functions to protect the body’s internal structures from external threats such as ultraviolet (UV) radiation, harmful chemicals, and pathogens. In addition, it is also responsible for the barrier function to prevent water loss and the sensory function to detect external changes; thus, it is susceptible to damage because it is directly exposed to the external environment [1,2]. The skin is composed of three layers: the epidermis, dermis, and subcutaneous fat layer, and each layer has its own structural and physiological functions. In the epidermis, keratinocytes play a protective role in the skin by minimizing the loss of moisture, heat, and other components. Melanocytes act as melanin production, Langerhans cells act as immune functions, and Merkel cells act as sensory functions [2,3]. The dermis is mainly composed of matrix components such as collagen, elastin and extrafibrillar matrix, proteoglycans, and glycoproteins, which are made by fibroblasts that gives the skin strength and elasticity [4,5].

Direct and chronic epidermal exposure to ultraviolet B (UVB) radiation in the epidermis induces overproduction of reactive oxygen species (ROS), including superoxide anions, hydrogen peroxide, singlet oxygen, and hydroxyl radicals, which destroy the antioxidant defense systems and finally cause oxidative stress [6,7,8,9,10,11]. UVB-induced oxidative stress leads to excessive melanin production in melanocytes and a decrease in hyaluronic acid production in keratinocytes, which play an important role in development of pigmentation and moisture loss in the skin [6,7]. In the dermis, UVB-induced oxidative stress stimulates the production of pro-inflammatory cytokines and protein degradation in the extracellular matrix by matrix metalloproteinases (MMPs) that cause skin wrinkle formation [8,9,10,11]. Therefore, several studies suggest that the antioxidant properties can prevent photoaging mediated by the suppression of UVB-induced oxidative stress.

In the present study, we investigated the effects of edible bird’s nest extract on melanogenesis, hyaluronic acid production, and wrinkle formation in the skin using in vitro and in vivo models. Edible bird’s nest is a nest made of salivary secretions and has long been used in traditional Chinese medicine. Although previous studies showed that edible bird’s nest contains sialic acid and is effective in brain development and various chronic inflammatory diseases, studies on their effects on skin health are still lacking [12,13,14]. We examined the moisturizing-related hyaluronic acid and sphingomyelin, the melanogenesis-related cAMP/protein kinase A (PKA)/cAMP-responsive binding protein (CREB)/melanocyte inducing transcription factor (MITF)/tyrosinase-related protein (TRP)/tyrosinase pathways, and elasticity-related c-Jun N-terminal kinase (JNK)/c-FOS/c-Jun/MMP and transforming growth factor-β receptor I (TGF-β RI)/small molecules against decapentaplegic homolog (Smad) pathways to understand the mechanisms underlying effects of edible bird’s nest extract on UVB-induced skin oxidative stress.

## 2. Materials and Methods

### 2.1. Edible Bird’s Nest Extract Preparation

**Figure 1:**
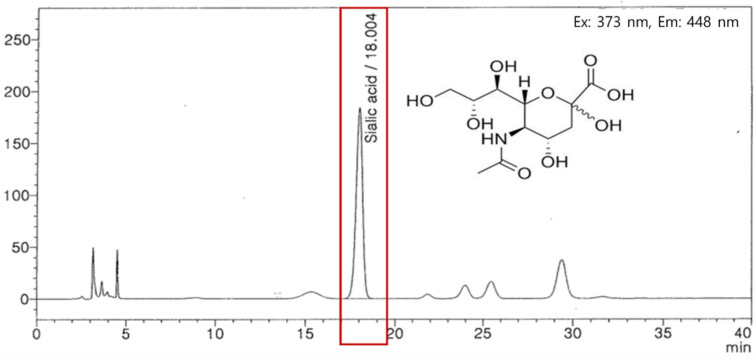
High-performance liquid chromatography analysis for sialic acid levels in BNE-PK.

Edible bird’s nest extract was obtained from Oryza Oil & Fat Chemical Co., Ltd. (Ichinomiya, Japan). Bird’s nests were ground and treated with powdered protease from *Bacillus amyloliquefaciens*. The extracts were filtrated, concentrated, mixed with maltodextrin, and dried (BNE-PK). We then analyzed the sialic acid levels in the BNE-PK using high-performance liquid chromatography. BNE-PK was standardized by 0.96–1.44% sialic acid (Figure 1) and stored in an air-tight container at −20 °C until use.

### 2.2. Animals and UVB Irradiation

SKH-1 hairless mice (five-week-old, male) were purchased from SaeRon Bio (Ui-wang, Korea) and housed in cages under automatically controlled temperature (22 ± 2 °C), humidity (about 50%), and lighting (12:12 h light/dark cycle) conditions. All mice were acclimatized for seven days before the experiment and then randomly divided into seven groups (eight animals/group): normal control (−UVB), control (+UVB), L-ascorbic acid (positive control 1; +UVB with dietary supplementation of L-ascorbic acid at 200 mg/kg/body weight [bw]), arbutin (positive control 2; +UVB with dietary supplementation of arbutin at 200 mg/kg/bw), BNE-PK 10 (+UVB with dietary supplementation of BNE-PK at 10 mg/kg/bw), BNE-PK 20 (+UVB with dietary supplementation of BNE-PK at 20 mg/kg/bw), and BNE-PK 40 (+UVB with dietary supplementation of BNE-PK at 40 mg/kg/bw).

Photoaging of skin was induced by UVB exposure of the dorsal skin in SKH-1 hairless mice, three times per week for 8 weeks, using a UVB lamp (Sankyo Denki Co., Yokohama, Japan). The minimal erythema dose (MED) was set at 150 mJ/cm^2^. The UVB dose schedule comprised UVB irradiation at 1 MED (150 mJ/cm^2^) in week 1, 2 MED (300 mJ/cm^2^) in week 2, 3 MED (450 mJ/cm^2^) in week 3, and 4 MED (600 mJ/cm^2^) in weeks 4 to 8. At the end of eight weeks, mice were sacrificed, and the dorsal skin and blood (by orbital venipuncture) were collected for analysis.

### 2.3. Measurement of Transepidermal Water Loss

Epidermal hydration of the dorsal skin surface was measured with Howskin (Seoul, Korea) under standardized conditions of 22–24 °C and 55–60% humidity.

### 2.4. Histological Observation

Dorsal skin tissues were fixed in 10% buffered formalin and then embedded in paraffin. The paraffin blocks were sliced into 5 μm sections, which were then stained with hematoxylin and eosin (H&E).

### 2.5. Measurement of Antioxidant Enzyme Activity in the Dorsal Skin

Dorsal skin tissues were collected from mice, and the activities of superoxide dismutase (SOD), catalase, and glutathione peroxidase (GPx) were measured using respective assay kits, superoxide dismutase 1 (SOD1) (Mouse) ELISA Kit, Catalase Activity Colorimetric/Fluorometric Assay Kit, and Glutathione Peroxidase Activity Colorimetric Assay Kit (BioVision Inc., Milpitas, CA, USA).

### 2.6. Cell Culture and Treatments

HaCaT cells and B16F10 cells were obtained from the American Type Culture Collection (ATCC; Manassas, VA, USA). The cells were cultured in Dulbecco’s minimal essential medium (Hyclone Laboratories, Logan, UT, USA) with 10% fetal bovine serum (Hyclone Laboratories, Logan, UT, USA), 100 mg/L penicillin–streptomycin (Hyclone Laboratories, Logan, UT, USA), and 2 mmol/L glutamine (Hyclone Laboratories, Logan, UT, USA). HaCaT cells were exposed to UVB (50 mJ/cm^2^) using a UVB lamp (5 Sankyo Denki G5T5 lamps, Sankyo Denki Co., Yokohama, Japan), followed by treatment with 100 µg/mL L-ascorbic acid or BNE-PK. Melanogenesis was induced in B16F10 cells using 250 µM isobutylmethylxanthine (IBMX), followed by treatment with 100 µg/mL arbutin or BNE-PK, for 72 h. The treated HaCaT cells and B16F10 cells were then harvested for further assays.

### 2.7. Protein Extraction and Western Blot Analysis

The dorsal skin tissues and cells were lysed using CelLytic MT cell lysis reagent (Sigma, St. Louis, MO, USA). Protein samples of 100 μg were separated using 10% Mini-PROTEAN^®^TGX™Precast Protein Gel (Bio-Rad Laboratories, Hercules, CA, USA) and electrotransferred onto polyvinylidene difluoride (PVDF) membranes (Bio-Rad Laboratories, Hercules, CA, USA). Membranes were blocked with 5% skimmed milk in Tris-buffered saline with 0.1% Tween 20 (TBST) for 1 h, then incubated for 12 h at 4 °C with antibodies against JNK, p-JNK, c-Fos, p-c-Fos, c-Jun, p-c-Jun, MMP-1, MMP-3, MMP-9, Smad3, p-Smad3, PKA, p-PKA, CREB, p-CREB, MITF, TRP-1, TRP-2, tyrosinase, HAS2, CerS4, and β-actin (1:1000) antibodies. All antibodies were purchased from Cell Signaling (Beverly, MA, USA). After incubation with the primary antibody, the membranes were incubated with a secondary antibody (anti-rabbit IgG HRP-linked antibody, 1:5000, Cell Signaling, Beverly, MA, USA) for 1 h at room temperature. The protein bands were detected with EzWestLumi Plus (ATTO, Tokyo, Japan) and developed using Ez-Capture II (ATTO). The bands were quantified using CS Analyzer 3.0 (ATTO).

### 2.8. Isolation of Total RNA and Reverse Transcription-PCR

Total RNA from dorsal skin tissues and cells was isolated using the RNeasy Mini kit (Qiagen, Valencia, CA, USA). Complementary DNA synthesis was performed using an iScript™ cDNA Synthesis Kit (Bio-Rad, Hercules, CA, USA). PCR amplification consisted of 40 cycles of 95 °C for 15 s, 58 °C for 15 s, and 72 °C for 30 s with SYBR Green PCR Master Mix (Bio-Rad) and primer pairs (Table 1). The data analysis was performed using 7500 System SDS software version 1.3.1 (Applied Biosystems, Foster City, CA, USA).

### 2.9. Measurement of Hyaluronic Acid and Sphingomyelin Levels

HaCaT cells were lysed, and hyaluronic acid and sphingomyelin levels were then determined using the Hyaluronic acid ELISA Kit (BioVision Inc., Milpitas, CA, USA) and Sphingomyelin Assay Kit (Abcam, Cambridge, UK), respectively, according to the manufacturer protocols.

### 2.10. Measurement of Intracellular Melanin and Glutathione (GSH) Contents

B16F10 cells were lysed and glutathione levels were determined using a Glutathione Assay Kit (BioVision Inc., Milpitas, CA, USA) according to the manufacturer’s protocols. B16F10 cells were lysed and then dissolved in 1 N NaOH, containing 10% dimethyl sulfoxide (DMSO), at 100 °C for 10 min; melanin content was then analyzed at 450 nm using an ELISA reader (iMark™ Microplate Absorbance Reader, Bio-Rad Laboratories, Hercules, CA, USA).

### 2.11. Measurement of Tyrosinase Activity, Nitric Oxide, and cAMP Levels

B16F10 cells and dorsal skin tissues were lysed, and tyrosinase activity and the levels of nitric oxide (NO) and cyclic AMP (cAMP) were then determined using the Tyrosinase Activity Assay Kit (Abcam, Cambridge, UK), Nitric Oxide Assay Kit (Abcam, Cambridge, UK), and cAMP ELISA Kit (Enzo Life Sciences, PA, USA), respectively, according to the manufacturer protocols.

### 2.12. Statistical Analysis

All data are presented as mean ± standard deviation (SD). Differences among groups were evaluated by one-way ANOVA and Duncan’s multiple range tests implemented in SPSS for Windows (SPSS PASW Statistic 22.0, SPSS Inc., Chicago, IL, USA). Differences were considered significant at *p* < 0.05.

## 3. Results

### 3.1. BNE-PK Suppressed Morphological and Histopathological Changes and Oxidative Stress in UVB-Irradiated Hairless Mice

**Figure 2:**
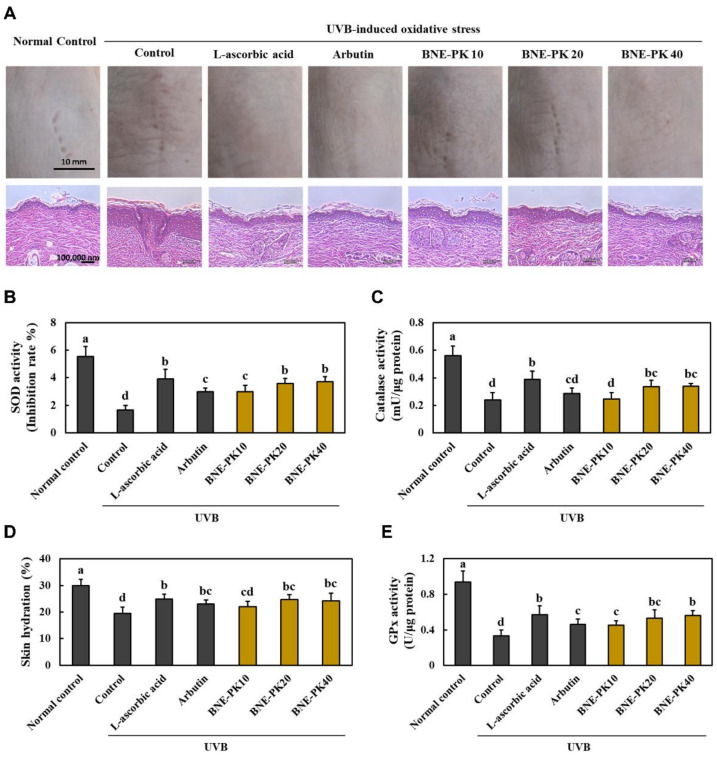
Effects of BNE-PK on morphological and histopathological changes (**A**), skin hydration (**B**), and antioxidant activities of SOD (**C**), catalase (**D**), and GPx (**E**) in the dorsal skin of ultraviolet B (UVB)-irradiated hairless mice. Normal control (−UVB), control (+UVB), L-ascorbic acid (positive control 1; +UVB with dietary supplementation of L-ascorbic acid at 200 mg/kg/body weight [bw]), arbutin (positive control 2; +UVB with dietary supplementation of arbutin at 200 mg/kg/bw), BNE-PK 10 (+UVB with dietary supplementation of BNE-PK at 10 mg/kg/bw), BNE-PK 20 (+UVB with dietary supplementation of BNE-PK at 20 mg/kg/bw), and BNE-PK 40 (+UVB with dietary supplementation of BNE-PK at 40 mg/kg/bw). Values are presented as mean ± SD. Different letters indicate a significant difference, with *p* < 0.05, as determined with Duncan’s multiple range test.

Figure 2A shows the results of morphological and histopathological changes, and Figure 2B shows the skin hydration in the dorsal skin of UVB-irradiated hairless mice. Dietary supplementation of L-ascorbic acid, arbutin, or BNE-PK attenuated UVB irradiation-induced morphological and histopathological changes including wrinkle formation, epidermal thickness, and irregularly shaped skin, and significantly increased skin hydration, compared to those in the control group (*p* < 0.05). In addition, the L-ascorbic acid, arbutin, and BNE-PK supplementation groups showed significant increase in the antioxidant activities of enzymes, including SOD, catalase, and GPx, compared to those in the control group (*p* < 0.05) (Figure 2C–E). These results indicated that BNE-PK supplementation effectively suppressed UVB irradiation-induced morphological and histopathological changes and oxidative stress in dorsal skin.

### 3.2. BNE-PK Increased Moisturizing and Elasticity Factors Activation and Decreased Melanogenesis Factor Activation in UVB-Irradiated Hairless Mice

We investigated the moisturizing, melanogenesis, and elasticity-related factors in the dorsal skin from UVB-irradiated hairless mice to confirm the molecular mechanism of BNE-PK’s effect on skin health.

**Figure 3:**
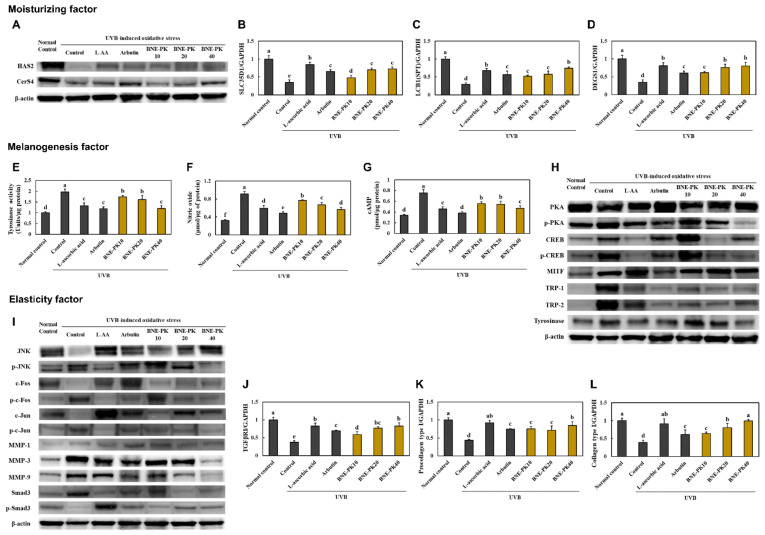
Effects of BNE-PK on protein expression of HAS2 and CerS4 (**A**), *SLC35D1* mRNA (**B**), *LCB1* mRNA (**C**), *DEGS1* mRNA (**D**), tyrosinase activity, (**E**) nitric oxide (**F**), cAMP (**G**), PKA/CREB/MITF/TRP-1/TRP-2/tyrosinase pathway (**H**), JNK/c-FOS/c-Jun/MMPs/Smad3 pathway (**I**), *TGF-β RI* mRNA (**J**), *procollagen Type I* mRNA (**K**), and *collagen Type I* mRNA (**L**) in the dorsal skin of ultraviolet B (UVB)-irradiated hairless mice. Normal control (−UVB), control (+UVB), L-ascorbic acid (positive control 1; +UVB with dietary supplementation of L-ascorbic acid at 200 mg/kg/body weight [bw]), arbutin (positive control 2; +UVB with dietary supplementation of arbutin at 200 mg/kg/bw), BNE-PK 10 (+UVB with dietary supplementation of BNE-PK at 10 mg/kg/bw), BNE-PK 20 (+UVB with dietary supplementation of BNE-PK at 20 mg/kg/bw), and BNE-PK 40 (+UVB with dietary supplementation of BNE-PK at 40 mg/kg/bw). Values are presented as mean ± SD. Different letters indicate a significant difference, with *p* < 0.05, as determined with Duncan’s multiple range test.

We found that that UVB-irradiated hairless mice supplemented with L-ascorbic acid, arbutin, and BNE-PK showed a significant increase in hyaluronic acid synthesis-related factors, protein expression of hyaluronic acid synthase 2 (HAS2), and mRNA expression of *SLC35D1* (UDP-glucuronic acid/UDP-N-acetylgalactosamine transporter), compared to those in control mice. In addition, L-ascorbic acid, arbutin, and BNE-PK supplementation to UVB-irradiated hairless mice increased the ceramide synthesis-related factors, protein expression of CerS4 (ceramide synthase 4), and mRNA expression of *LCB1* (Srine palmitoyltransferase 1) and *DEGS1* (delta 4-desaturase, sphingolipid 1), compared with that in the control group (*p* < 0.05) (Figure 3A–D, Figure S1).

In the results of melanogenesis-related factors, L-ascorbic acid, arbutin, and BNE-PK supplementation in UVB-irradiated hairless mice decreased the levels of tyrosinase activity, NO, and cAMP, compared to those in control mice (*p* < 0.05) (Figure 3E–G). Moreover, the expressions of PKA/CREB/MITF/TRP-1/TRP-2/tyrosinase pathway proteins were decreased in the L-ascorbic acid, arbutin, and BNE-PK supplementation groups, compared to those in control mice (Figure 3H, Figure S1). Figure 3I and Figure S1 show that UVB-irradiated hairless mice supplemented with L-ascorbic acid, arbutin, and BNE-PK showed a significant decrease in the protein expression of the JNK/c-FOS/c-Jun/MMPs pathway and a significant increase in Smad3 phosphorylation. In addition, the L-ascorbic acid, arbutin, and BNE-PK supplementation groups showed significant increase in the mRNA expression of *TGF-β RI*, *procollagen Type I*, and *collagen Type I*, compared to those in the control group (*p* < 0.05) (Figure 3J–L).

### 3.3. Effect of BNE-PK Treatment on Moisturizing Factors in UVB-Irradiated HaCaT Cells

**Figure 4:**
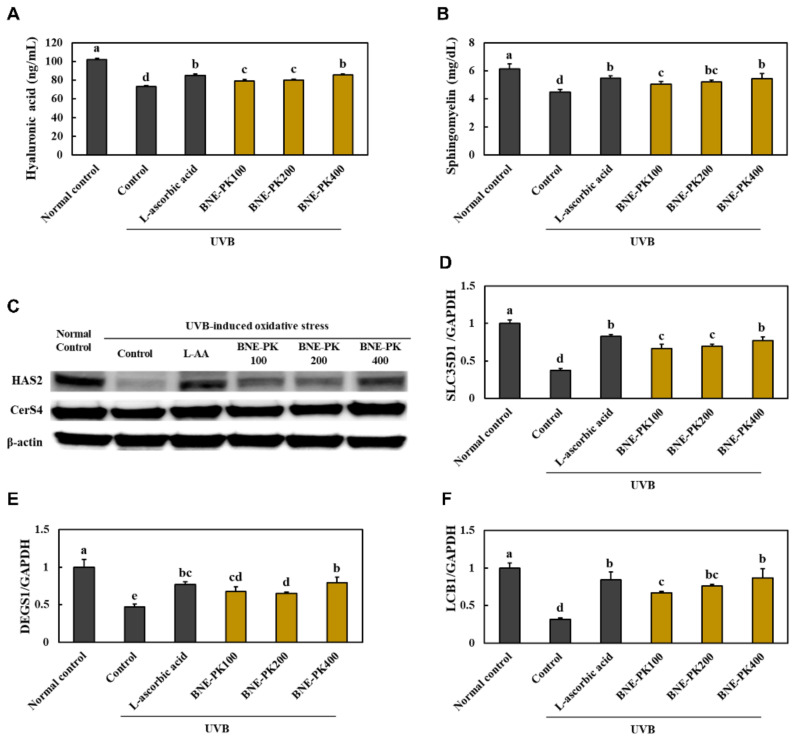
Effects of BNE-PK on the levels of hyaluronic acid (**A**) and sphingomyelin (**B**), protein expression of HAS2 and CerS4 (**C**), and mRNA expression of *SLC35D1* (**D**), *DEGS1* (**E**), and *LCB1* (**F**) in ultraviolet B (UVB)-irradiated HaCaT cells. Normal control (NC; −UVB), control (C; +UVB), L-ascorbic acid (positive control; L-ascorbic acid treatment at 100 μg/mL), and BNE-PK treatment at various concentrations (100, 200, and 400 μg/mL). Values are presented as mean ± SD. Different letters indicate a significant difference, with *p* < 0.05, as determined with Duncan’s multiple range test.

We found that that UVB-irradiated HaCaT cells treated with L-ascorbic acid and BNE-PK showed a significant increase in the levels of hyaluronic acid and sphingomyelin, compared with that in the control group (*p* < 0.05) (Figure 4A,B). L-ascorbic acid and BNE-PK treatment to UVB-irradiated HaCaT cells increased the hyaluronic acid synthesis-related factors, protein expression of HAS2, and mRNA expression of *SLC35D1*, compared to those in control mice. Further, L-ascorbic acid and BNE-PK treatment to UVB-irradiated HaCaT cells increased the ceramide synthesis-related factors, protein expression of CerS4, and mRNA expression of *LCB1* and *DEGS1*, compared with that in the control group (*p* < 0.05) (Figure 4C–F, Figure S2) shows that UVB-irradiated hairless mice supplemented with L-ascorbic acid). Therefore, we suggest that BNE-PK can directly activate moisturizing factors in keratinocytes.

### 3.4. Effect of BNE-PK Treatment on Melanogenesis Factors in IBMX-Treated B16F10 Cells

**Figure 5:**
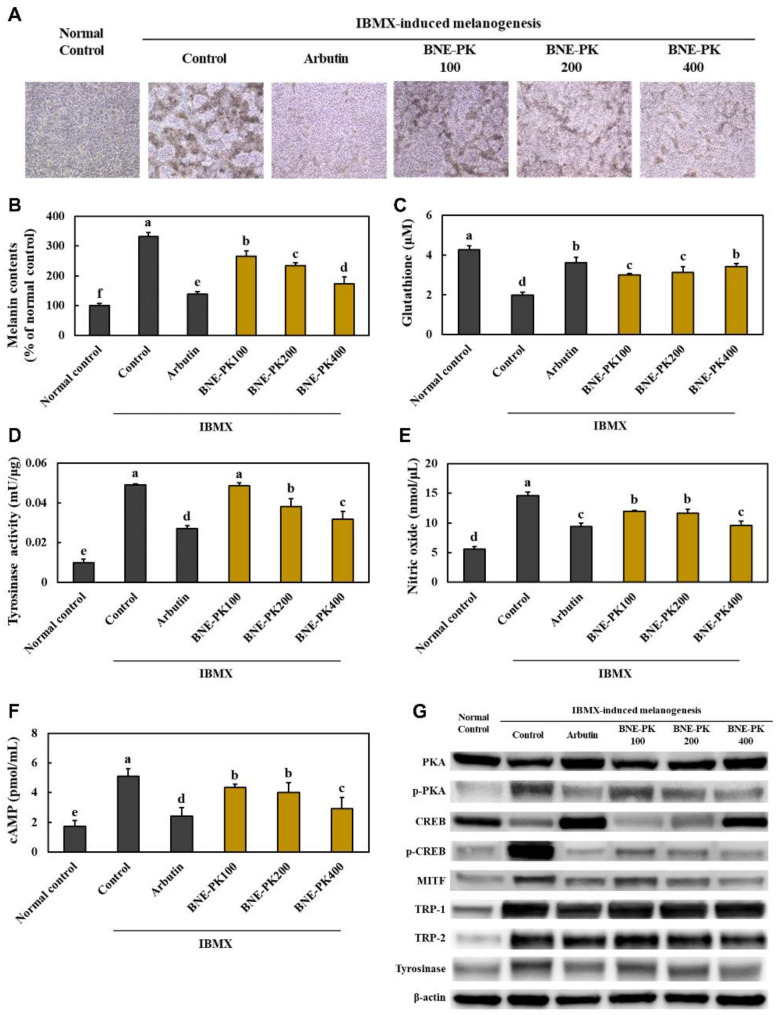
Effects of BNE-PK on the melanin contents (**A**,**B**), glutathione (**C**), tyrosinase activity (**D**), nitric oxide (**E**), cAMP (**F**), and PKA/CREB/MITF/TRP-1/TRP-2/tyrosinase pathway (**G**) in IBMX-treated B16F10 cells. Normal control (NC; −IBMX), control (C; + IBMX), arbutin (positive control; arbutin treatment at 100 μg/mL), and BNE-PK treatment at various concentrations (100, 200, and 400 μg/mL). Values are presented as mean ± SD. Different letters indicate a significant difference, with *p* < 0.05, as determined with Duncan’s multiple range test.

We found that the melanin contents were significantly decreased in the arbutin and BNE-PK-treated groups compared with those in the control group (*p* < 0.05) (Figure 5A,B). Moreover, treatment with arbutin and BNE-PK significantly decreased the levels of tyrosinase activity, nitric oxide, and cAMP, and significantly increased the glutathione levels in the IBMX-treated B16F10 cells (*p* < 0.05) (Figure 5C–F). Arbutin and BNE-PK treatment to IBMX-treated B16F10 cells decreased the expression of PKA/CREB/MITF/TRP-1/TRP-2/tyrosinase pathway proteins compared with that in the control group (*p* < 0.05) (Figure 5G, Figure S2). These results indicated that BNE-PK directly suppressed melanogenesis in melanocytes.

## 4. Discussion

Cutaneous aging induced by complex biological phenomena consists of both intrinsic and extrinsic factors. Intrinsic aging is genetically determined and inevitable due to physiological aging processes, while extrinsic aging is engendered by external factors such as temperature, pollution, and UV irradiation [15]. Skin aging caused by UV irradiation is called photoaging, which is characterized by deep wrinkles, pigmentation, dry and rough skin, and loss of moisture and elasticity [16,17]. It is well accepted that chronic UVB irradiation of the skin generates excessive ROS, which promotes the secretion of pro-inflammatory cytokines and irregular melanin production in the epidermis [9]. Secreted pro-inflammatory cytokines from the epidermis trigger nuclear factor kappa-light-chain-enhancer of activated B cells (NF-κB) pathway activation that induces progressive inflammation and MMP-induced proteolysis via JNK pathway activation in the dermis [9,10]. In the present study, we investigated the effects of BNE-PK in UVB irradiation-induced oxidative stress and photoaging though confirming the effect on hyaluronic acid production, melanogenesis, and wrinkle formation. Our results showed that BNE-PK suppressed wrinkle formation, irregularly shaped skin, hydration loss, and decreased antioxidant activities in UVB-irradiated hairless mice, indicating that BNE-PK protects the skin against UVB irradiation by exerting antioxidants.

Hyaluronic acid is distributed widely throughout connective and epithelial tissues, and half of this is found in the skin tissue. Hyaluronic acid in the skin plays a key role in physiological water balance regulation; thus, up-regulation of hyaluronic acid levels help maintain skin moisturizing [18,19]. In the study by Dai et al., repetitive UVB irradiation induced loss of hyaluronic acid by transcriptional down-regulation of HAS [6]. Our findings showed that BNE-PK increased hyaluronic acid synthesis-related HAS2 and mRNA expression of *SLC35D1*, ceramide synthesis-related CerS4 protein expression, and mRNA expression of *LCB1* and *DEGS1* in both in vitro and in vivo models using UVB irradiation. These findings indicate that BNE-PK protects the hydration loss by up-regulation of hyaluronic acid synthesis during UVB irradiation.

Moreover, our data showed that BNE-PK suppressed melanogenesis by inhibiting the cAMP/PKA/CREB/MITF/TRP-1/TRP-2/tyrosinase pathway, tyrosinase activation, and glutathione synthesis. Although the melanocytes produce melanin to protect the skin from UV radiation, excessive melanin production by chronic UV radiation is associated with the development of several skin diseases [20]. During UVB irradiation in the skin, NO accumulation and activation of the cAMP/PKA/CREB pathway stimulated the expression of MITF, which regulates the transcription of tyrosinase and eumelanin synthesis, and increased glutathione stimulates synthesis of pheomelanin [21,22,23]. Therefore, we suggest that UVB irradiation-induced melanogenesis in melanocytes can be inhibited by BNE-PK treatment.

Collagen type I is the most abundant collagen in the skin and provides tensile strength and stability to the dermis [24]. UVB irradiation inhibits SMAD3 phosphorylation by down-regulation of TGF-βRI and stimulates phosphorylation of JNK, c-Jun, and c-Fos, which cause MMPs-mediated collagen degradation [25,26]. We showed that BNE-PK attenuated the JNK/c-FOS/c-Jun/MMP pathway activation and stimulated the TGF-βRI/SMAD3/pro-collagen type I pathway activation during UVB irradiation in the in vivo model.

Sialic acid is the major carbohydrate found in edible bird’s nest and found in several tissues and fluids in humans. Ogasawara et al. [27] demonstrated the antioxidant role of sialic acid as a hydroxyl radicals scavenger, and Wang et al. [28] found that sialic acid from edible bird’s nest has the effects of scavenging DPPH radicals and hydroxyl radicals. Several studies demonstrated sialic acid is essential for brain function, immune function, and cell proliferation and repair [12,29,30], but its effects on skin health remain unknown. Although we have not confirmed the effect of sialic acid from edible bird’s nest on anti-photoaging, these previous studies suggest the potential role of sialic acid in the effect of BNE-PK. Saengkrajang et al. [31] analyzed the chemical composition of edible bird’s nest and found that it included 61.0–66.9% protein and 25.4–31.4% carbohydrates. Additional studies on the nutritional composition of sialic acid from BNE-PK and on the clinical effects are required to verify the effective levels and molecular mechanisms in anti-photoaging.

## 5. Conclusions

We demonstrated that standardized edible bird’s nest extract prevented UVB irradiation-mediated oxidative stress and photoaging in the skin using in vitro and in vivo models. BNE-PK increased skin hydration by hyaluronic acid synthesis and suppressed melanogenesis by down-regulation of the cAMP/PKA/CREB/MITF/TRP-1/TRP-2/tyrosinase pathway and wrinkle formation by down-regulation of the JNK/c-FOS/c-Jun/MMP pathway during UVB irradiation-mediated oxidative stress. Based on these findings, we suggest that supplementation with BNE-PK may be useful for preventing skin photoaging.

## Figures and Tables

**Table 1:** Primer sets used for real-time PCR.

Gene	Accession Number	Sequence
*Procollagen Type I* (M)	NM_008788.2	F 5′-TTA CGT GGC AAG TGA GGG TTT-3′
		R 5′-TGT CCA GAT GCA CTT CTT GTT TG-3′
*Collagen Type I* (M)	NM_007742.4	F 5′-GAC CGT TCT ATT CCT CAG TGC AA-3′
		R 5′-CCC GGT GAC ACA CAA AGA CA-3′
*TGF-β RI* (M)	AF271072.1	F 5′-CAT CCT GAT GGC AAG AGC TAC A-3′
		R 5′-TAG TGG ATG CGG ACG TAA CCA-3′
*SLC35D1* (M)	AB117931.1	F 5′-TTC CTC ATC GTG CTG GTC AA-3′
		R 5′-TTG GTG AGG GAA AAC CGT ATG-3′
*LCB1(SPT)* (M)	AF003823.1	F 5′-AGC GCC TGG CAA AGT TTA TG-3′
		R 5′-GTG GAG AAG CCG TAC GTG TAA AT-3′
*DEGS1* (M)	NM_007853.5	F 5′-CCG GCG CAA GGA GAT CT-3′
		R 5′-TGT GGT CAG GTT TCA TCA AGG A-3′
*GAPDH* (M)	NM_001289726.1	F 5′-CAT GGC CTT CCG TGT TCC TA-3′
		R 5′-GCG GCA CGT CAG ATC CA-3′
*SLC35D1* (H)	AB044343.1	F 5′-TCC TGA TCG TGG TGG TGA ATA A-3′
		R 5′-ACA CAT AGT GAG GAG GGA AAT CTG T-3′
*LCB1(SPT)* (H)	BC068537.1	F 5′-CCA TGG AGT GGC CTG AAA GA-3′
		R 5′-CTG ACA CCA TTT GGT AAC AAT CCT A-3′
*DEGS1* (H)	NM_003676.4	F 5′-GCT GAT GGC GTC GAT GTA GA-3′
		R 5′-TGA AAG CGG TAC AGA AGA ACC A-3′
*GAPDH* (H)	NG_007073.2	F 5′-CCC CAC ACA CAT GCA CTT ACC-3′
		R 5′-TTG CCA AGT TGC CTG TCC TT-3′

## Data Availability

The data is contained within this article and supplementary file.

## Supplementary Materials

The following are available online at https://www.mdpi.com/article/10.3390/antiox10091452/s1, Figure S1. Quantification of the western blot bands relative expression in Figure 3, Figure S2. Quantification of the western blot bands relative expression in Figure 4 and Figure 5.
