# Supplementary material for: Standardized Edible Bird’s Nest Extract Prevents UVB Irradiation-Mediated Oxidative Stress and Photoaging in the Skin
Source: Antioxidants (Basel). 2021 Sep 13;10(9):1452. doi: 10.3390/antiox10091452 (PMC8470287; doi:10.3390/antiox10091452)
Supplement: Supplementary file 1 [file antioxidants-10-01452-s001.zip › antioxidants-1326892-supplementary.pdf]

Quantifications of Fig. 3A bands

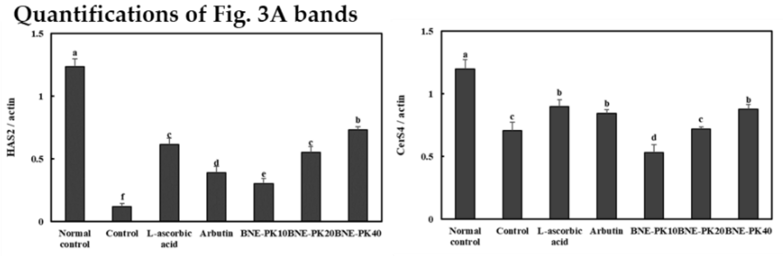

Quantifications of Fig. 3H bands

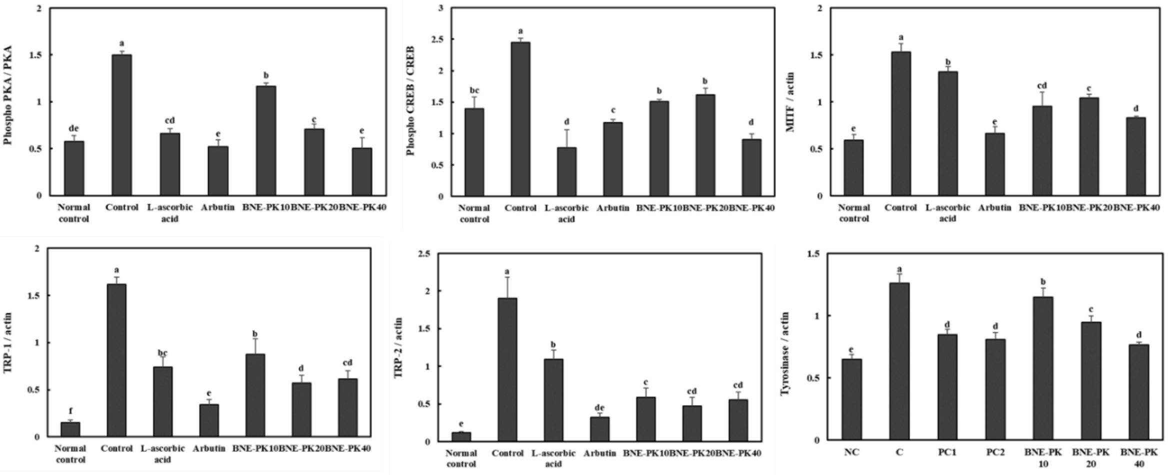

Quantifications of Fig. 3I bands

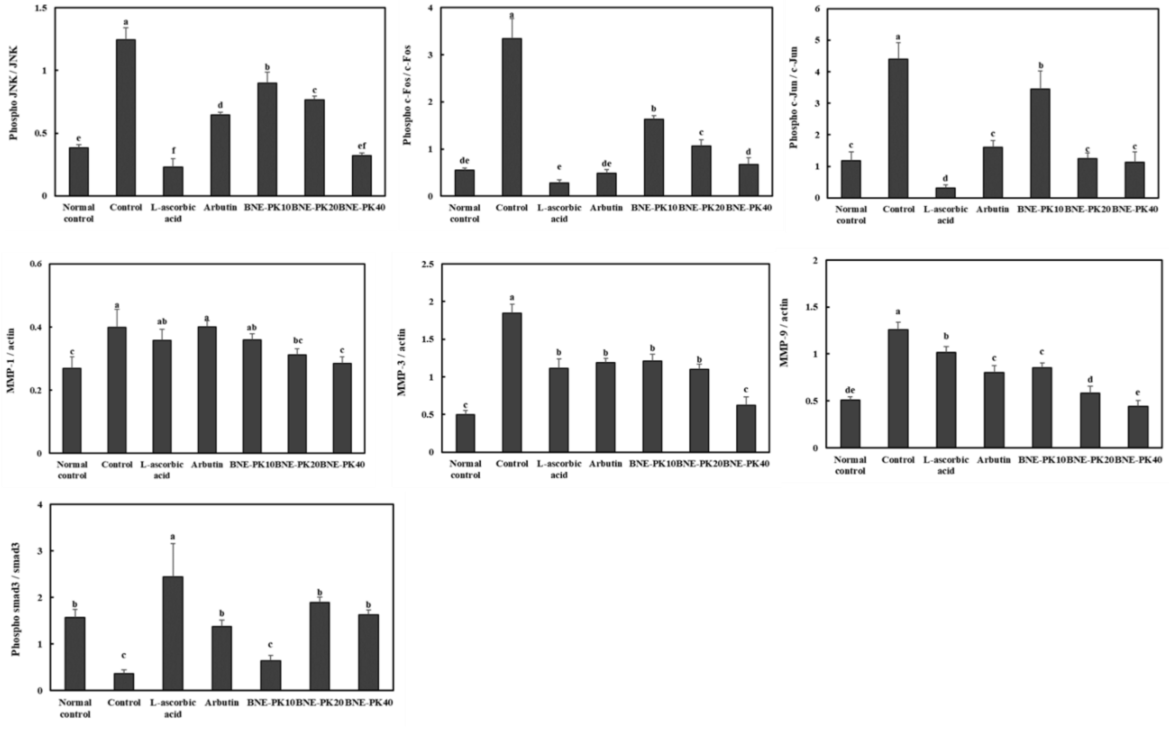

Sup 1. Quantification of the western blot bands relative expression in Figure 3.

Quantifications of Fig. 4C bands

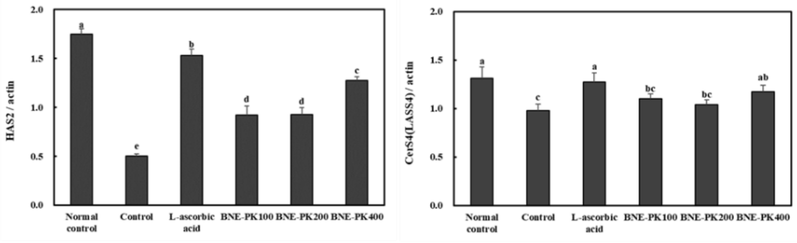

Quantifications of Fig. 5G bands

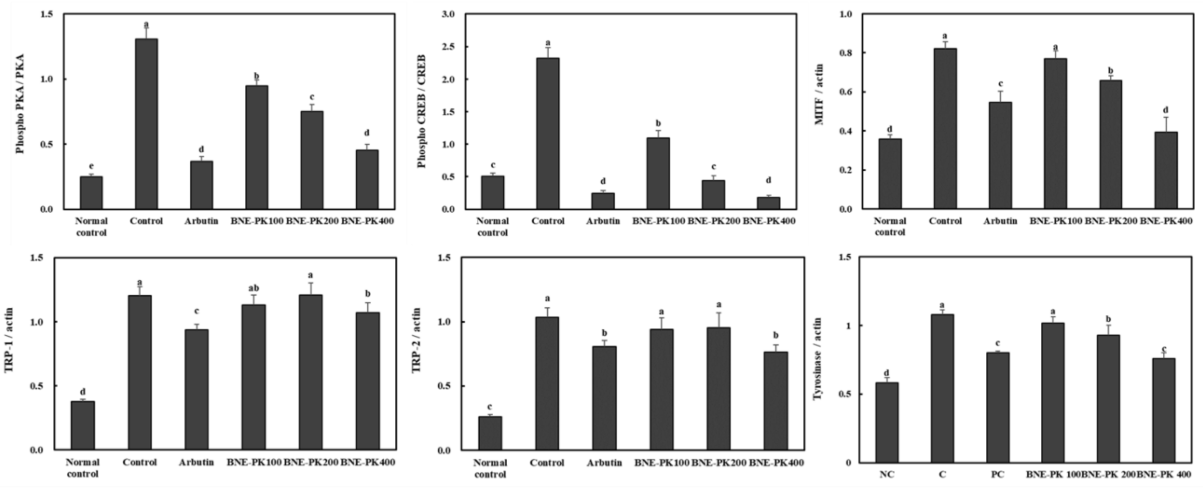

Sup 2. Quantification of the western blot bands relative expression in Figure 4 and 5.
